# Flexibility quantification in households: a swiss case study

**DOI:** 10.1186/s42162-020-00126-4

**Published:** 2020-10-28

**Authors:** Yousra Sidqi, Pierre Ferrez, Dominique Gabioud, Pierre Roduit

**Affiliations:** University of Applied Sciences Western Switzerland, Route du Rawil 47, Sion, 1950 Switzerland

**Keywords:** Demand Side Management (DSM), Demand Response (DR), Residential Flexibility

## Abstract

In this paper, a thorough analysis of quantification of the heating appliances’ flexibility provided by 200 households located in the Sion area (Switzerland) is presented. An extended evaluation of the available flexibility throughout the year as well as a correlation analysis between the outside temperature and flexibility is performed. The influence of pooling households in the prediction process is assessed. The impact of cutting the power to heating appliances and the incurred rebound effect are also described.

## Introduction

The increasing penetration rate of new non-dispatchable intermittent renewables (Photovoltaic (PV), wind) puts at stress the electrical system: balancing production and consumption becomes more challenging and parts of the grid can become congested at peak times. Demand management, which aims at shaping consumption to fit needs of the electrical system, is one of the available means to relieve this stress. The residential sector represents about 30% of the total electricity consumption in Europe(Bertoldi and Atanasiu 2007) and is therefore an interesting target for demand management. Demand management programs are known as Demand Response (DR) programs. Several research and experimentation projects have investigated and demonstrated demand management in the residential sector (Fernández Aznar et al. 2019; Garbi et al. 2019; Schröder et al. 2018; Jacobsen et al. 2015).

The first section of this paper describes the state of the art regarding harvesting and analyzing flexibility in households. The second section presents the results of the quantification of available flexibility in households as well as the influence of pooling households to better predict their flexibility.

## Households flexibility

Flexibility potential in households is usually defined as the capacity to increase, decrease, or shift the consumption of domestic appliances over a period of time.

Flexibility analysis in households was conducted in Belgium (D’hulst et al. 2015), where smart appliances were installed in 239 households for three years. The appliances consisted in washing machines, dishwashers, tumble dryers, electric hot water tanks, and electric vehicles. The project showed that consumption of all wet appliances (e.g. washing machines, dishwashers, tumble dryers) in Belgium can be increased to a maximum of 2GW during 30 minutes and decreased to a low value of 300 MW at 10 p.m. in the weekend for 15 minutes. This represents an average maximal increase of 430 W and a maximum decrease of 65 W per household. Kaschub et al. (2016) explored the effects of increasing solar storage on Demand Side Management (DSM) in Germany. This study showed how a decrease in electricity demand, due to self-consumption, could lead to an increase in electricity price and negatively affect customers without flexible loads or self-generation units. Nicholls et al. (2015) studied flexibility in households in a qualitative way as they showed the influence of having children on the consumption and flexibility potential of a household. The analysis revealed how a family peak period related to institution arrangements, such as school times, working hours and outside-school activities.

Several studies focused on modelling the flexibility of building to allow better prediction and develop control strategies (Chen et al. 2019; Stinner et al. 2016; De Coninck and Helsen 2016; Finck et al. 2018; Arteconi et al. 2019; Zhou and Cao 2020). Here, we define flexibility quantification as a statistical overview of flexible energy in households based on real data.

## Quantification of flexibility

Within the framework of the H2020 GOFLEX project, The local Distribution System Operator (DSO) Enérgies Sion Réégion (ESR) has teamed up with the Institute of Sustainable Energy of the University of Applied Sciences and Arts Western Switzerland (HES-SO) to deploy an integrated solution, which contributes in the cost-effective use of demand response in distribution grids and increase of the available flexibility of loads/generation included in demand response schemes. The solution deployed in the area of Sion aims at using DSM to minimise the imbalance for the ESR’s sub-balancing group, thus lowering corrective costs, and to reduce peak loads on the distribution grid, thus reducing the need of upgrading the infrastructure in areas where decentralized PV production is increasing.

The possibility of storing energy in the form of heat makes space heating and hot water systems particularly interesting for flexibility purposes. A given volume of water is stored in a tank and preheated for later use. There is therefore a high potential for such loads in terms of consumption shift over time. Flexibility is defined by two aspects: the quantity of energy that could be shifted and for how long it would be possible.

In this section, the flexibility of heating appliances in 194 households, where the GOFLEX solution was installed, is assessed. The consumed power is quantified and the share of both heating needs, namely space heating and hot water heating, is computed.

The consumption profiles of households are computed using data collected between October 2018 and May 2020, which allows a data based quantification of the available flexible energy. They are then confronted to outside temperatures to better understand the correlation between these two measures. The quantification is conducted over pools of different sizes. Having bigger sized pools allows a better prediction accuracy, whereas working with smaller pools enables more spatial separation to help solving local congestions.

The DSO’s business case, due to local market restrictions, is to be able to get advantage of the available flexibility within a period of 1 to 2 hours. Here, cut tests of 1 and 2 hours were conducted on heat pumps to evaluate the restart behavior, while making sure not to alter the consumer’s comfort.

### Available energy

**Fig. 1:**
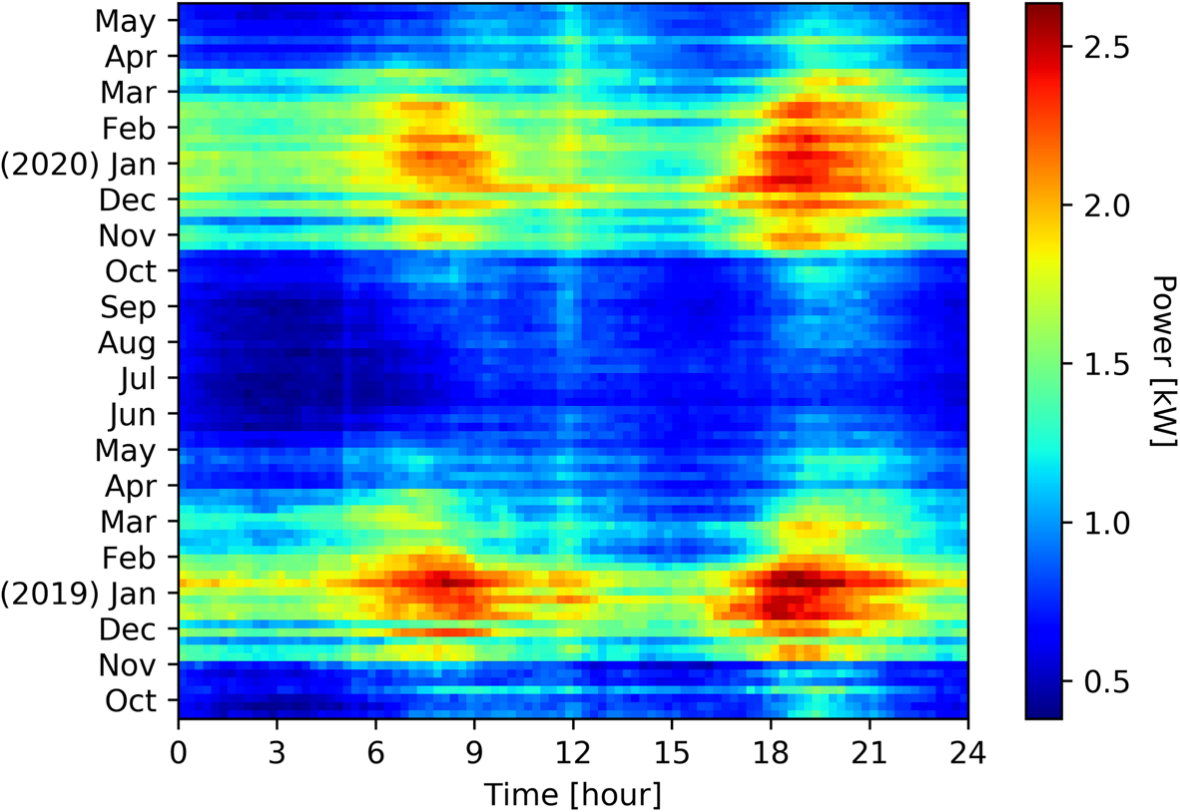
Weekly average total consumption per quarter of house of 194 households from October 2018 to May 2020. The heating and lighting influence in winter is clearly visible, as well as cooking at noon

**Fig. 2:**
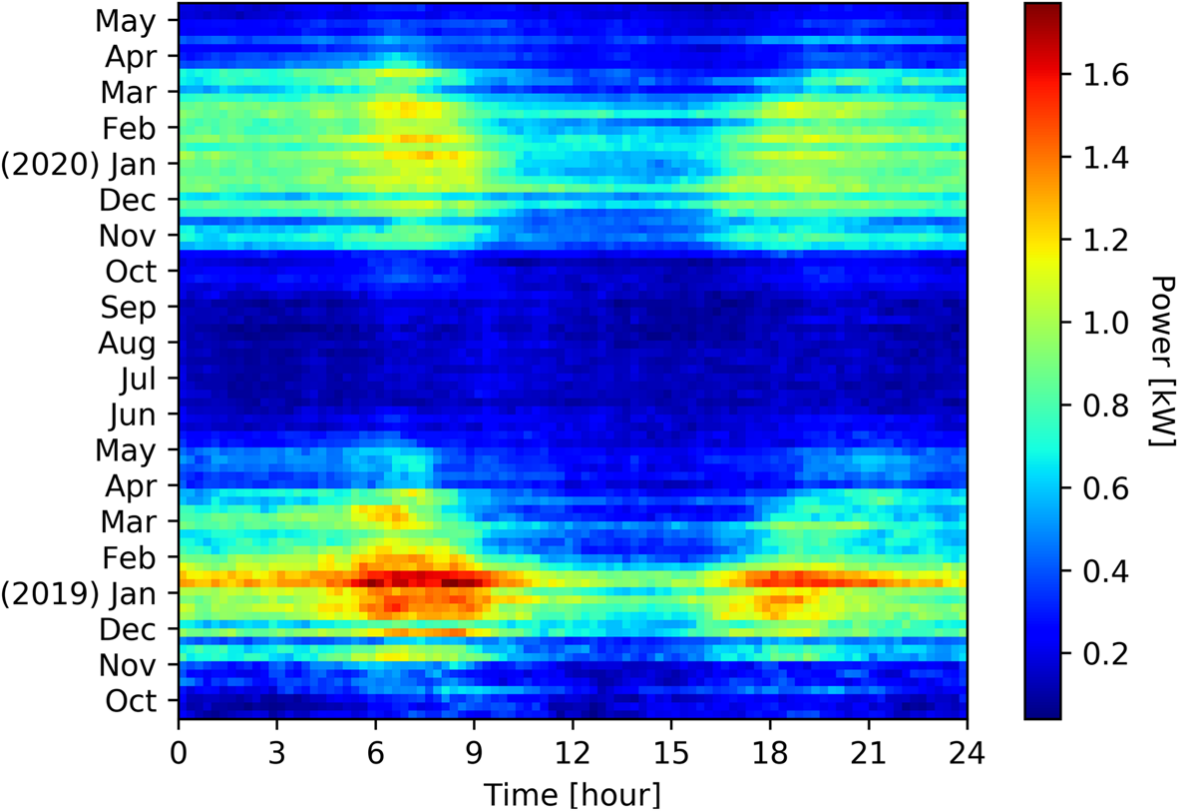
Weekly space heating consumption per quarter of house of 194 households from October 2018 to May 2020. Influence of the cold season can clearly be identified. Moreover, we can observe that winter 18-19 was colder as 19-20

**Fig. 3:**
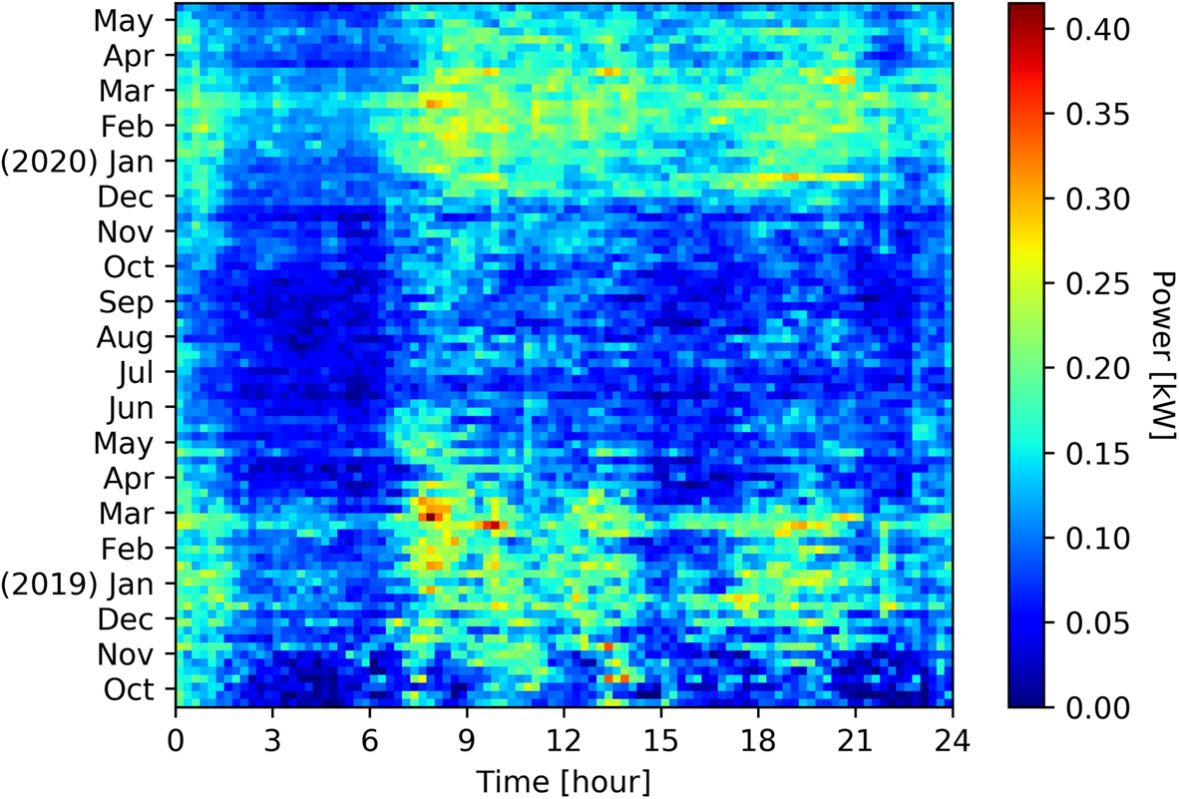
Weekly average hot water consumption per quarter of house of 194 households from October 2018 to May 2020. Consumption is quite regular, even if morning, evening, and cold induces an increase of the consumption

**Fig. 4:**
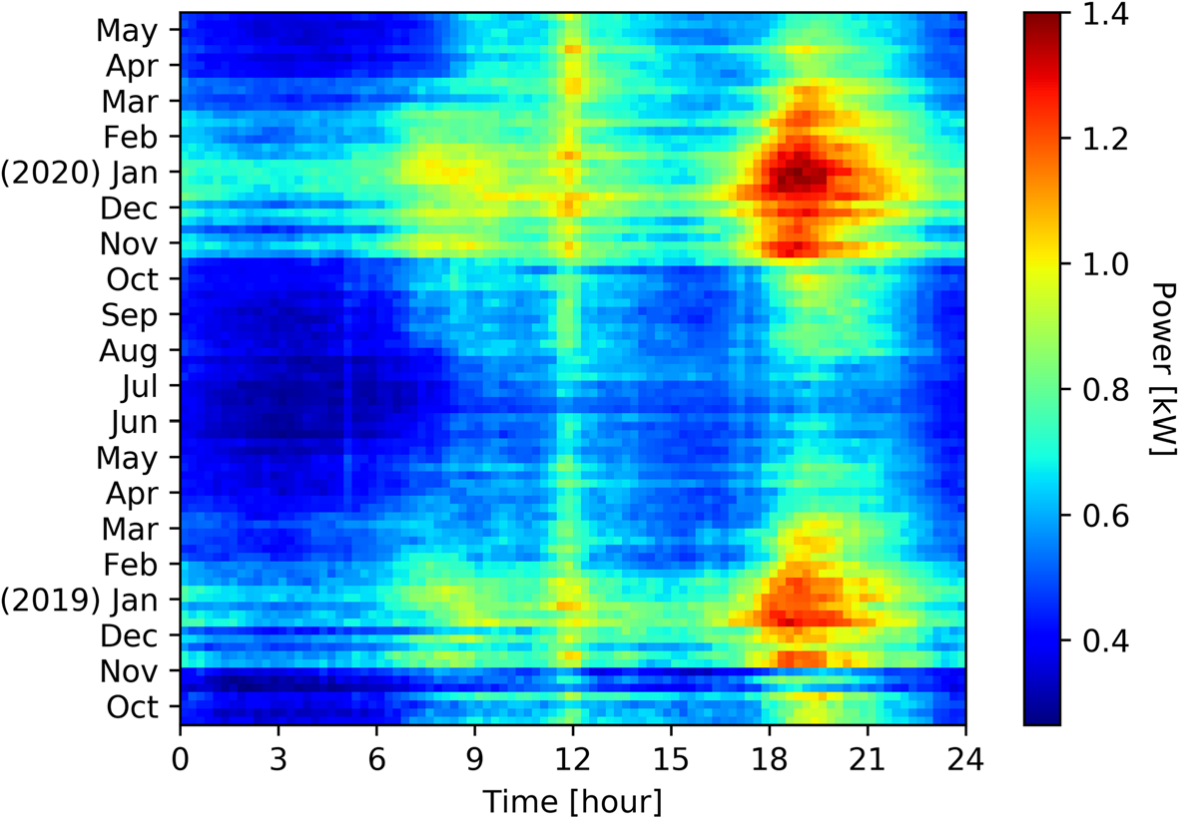
Weekly house appliances consumption per quarter of house of 194 households from October 2018 to May 2020 minus heating devices. Effect of more lighting in winter in the morning and evening can easily be spotted, as well as cooking at lunch time

Figure 1 shows the average total consumption per week and per quarter of hour of 194 households from October 2018 to May 2020. For the same households and the same time window, Fig. 2 shows the average space heating consumption, Fig. 3 shows the average hot water consumption, and Fig. 4 shows the average consumption of all the other appliances.

The power consumption for heating and hot water represents a big share of the total consumption during winter season. We observe two major peaks of consumption in the morning and afternoon where household activities (morning/evening showers, heating) are taking place while around the middle of the day this consumption drops as people are less likely to be in their homes in addition to the compensation of PV panels.

In Valais(Switzerland), there are strong seasonal temperature fluctuations which is the reason why consumption habits in summer are very different from those in winter. The resulting average consumption is thus greatly varying, from close to 0W in the summer up to more than 1.6 kW during the cold night of the winter season. The daily average temperatures range from -4 ^∘^C in January to 27 ^∘^C in July. During the heating season, the heating systems are used to keep the room temperature at around 22 ^∘^C. The period depends heavily on the location of the buildings, but usually lasts from September to May. During the heating season, electricity consumption increases in a brutal fashion as shown in Fig. 2. This means more loads can be used for DSM.

The power required for hot water supply follows the hot water requirement. In the morning and in the evening, when most people are taking showers, a higher consumption is recorded. There is also a small increase during the lunch break. During the spring season of 2020, there is an increase in terms of consumption compared to the same period during the previous year, which could be explained by the Covid-19 health crisis. People were mostly working remotely and children were off school during this period, which led to a shift in consumption behavior with regard to hot water as shown in Fig. 3.

Figure 4 shows the behavior of consumers outside of heating appliances. There is a constant behavior all year around midday, which corresponds to lunch break: cooking, dish washing. During winter, there is a higher consumption in early morning and evening, which corresponds mostly to lighting. The evening consumption is higher due to the concentration of activities requiring electricity: light, TV, cooking, etc. This part of the consumption is considered as too complicated to be shifted and as to be put in comparison with the heating appliances consumption These four Figs. (1, 2, 3, 4) clearly show the energy potential of heating appliances, even if this potential is greatly varying during the seasons and during the day.

### Heating vs temperature

The energy consumption of space heating and hot water appliances depends on numerous factors. In particular, seasonal changes have a large impact on the energy consumption of space heating appliances. Space heating appliances show their maximum consumption between December and February, but are mostly switched off between May and October.

**Fig. 5:**
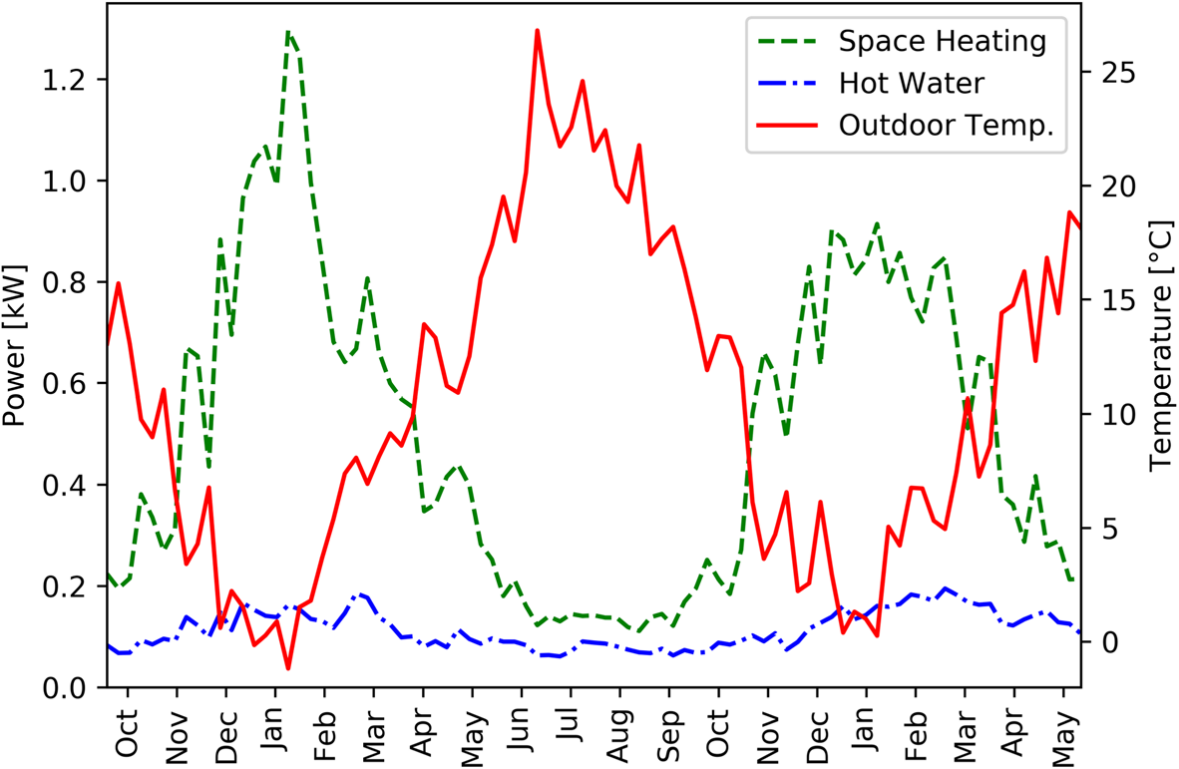
Weekly average outdoor temperature in Sion and weekly average consumed power of both space heating and hot water appliances between October 2018 and May 2020

Figure 5 shows the weekly average consumed power of both space heating and hot water appliances between October 2018 and May 2020. Figure 5 also shows the weekly average outdoor temperature in Sion for the same period. The temperature of the weather station of Sion (482 m a.s.l.) was used as a reference, even if the households are located in a broader area with some of them being located at more than 1000 m a.s.l.. The power consumed by hot water appliances is not constant but only ranges from about 60 W in summer to almost 200 W in Winter. As expected, the maximum power consumed by space heating appliances happens between December and February, corresponding to the lowest recorded temperature in the area. Between June and September, the hottest months of the year, Fig. 5 shows a non-zero minimal power consumption (slightly over 100 W).

**Fig. 6:**
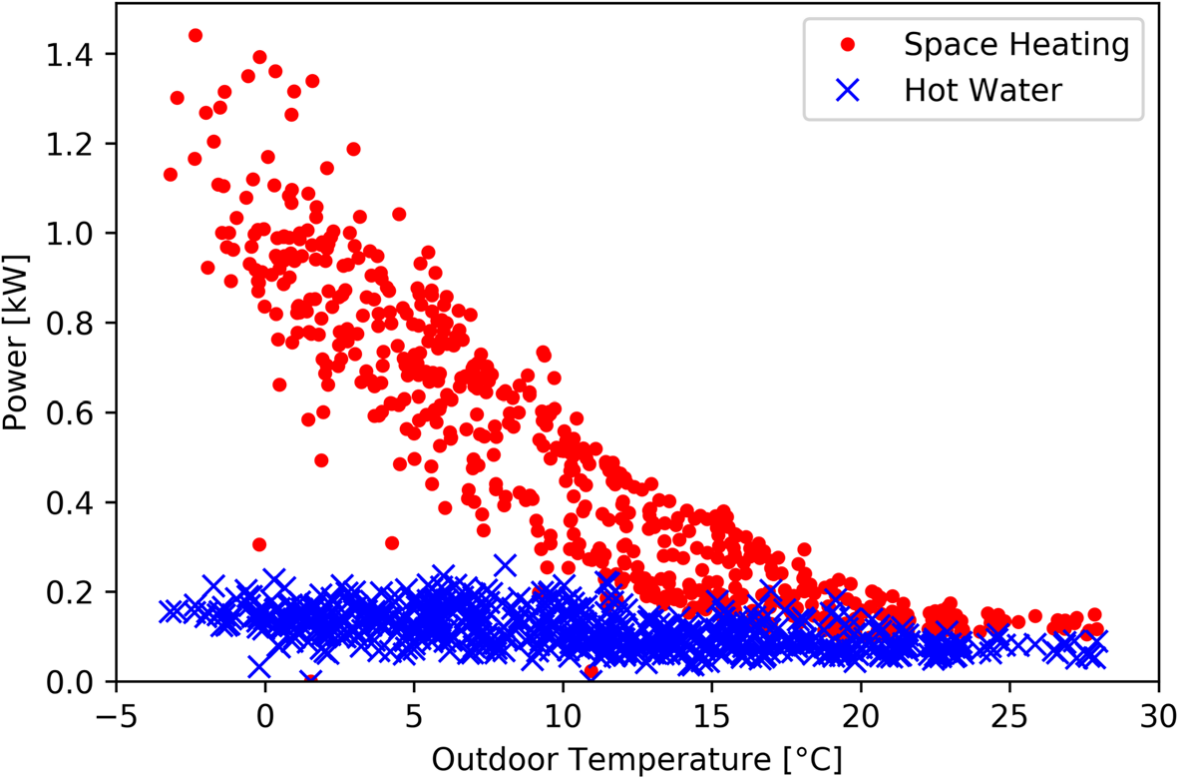
Average power consumption of the hot water and space heating appliances as a function of the outdoor temperature for every day between October 2018 and May 2020

Outside this hot period, the space heating power curve and the outdoor temperature curve seem well anti-correlated. Figure 6 shows the average power consumption of the hot water and space heating appliances are as a function of the outdoor temperature for every day between October 2018 and May 2020 and illustrates this anti-correlation. Figure 6 shows a clear decrease of space heating power with increasing outdoor temperature. Above 20 ^∘^C, the space heating power becomes constant but not null. This could be explained by the fact that the measured power also include the power required by circulation pumps or other non-heating components of the heating systems that were not shut down for the summer. It could also be explained by the fact that some of the monitored households are located in higher altitude and are thus experiencing lower outdoor temperatures. Their heating systems are still running and therefore consuming energy in summer.

Figure 6 also shows the slight decrease of hot water power with increasing outdoor temperature mentioned above. This could be explained by the fact that the cold water entering the system is colder in winter than in summer and therefore requires more energy to be heated up. It could also be explained by the simple fact that people might take warmer showers or baths in winter.

### Influence of pooling households on the predictability of the load

Given their high power consumption (a few kW), space heating and hot water appliances are extremely interesting candidates for flexibility purposes. However, the energy consumption of space heating and hot water appliances depends on numerous factors such as the residents’ habits and behaviour, the weather and the control systems. Indeed, these appliances are typically consuming high power levels a few times a day, and are off the rest of the time. This consumption is therefore very sporadic and its prediction for a given time is definitely not an easy task.

To fully benefit from the flexibility, we need to be able to predict with reasonable accuracy the power consumption at a future point in time. Pooling the households together should benefit the prediction of the load, in line with the Central Limit Theorem establishing that, in some situations, the normalized sum of independent random variables tends toward a normal distribution even if the original variables themselves are not normally distributed. The following part will thus focus on the analysis of the influence and benefit of pooling.

**Fig. 7:**
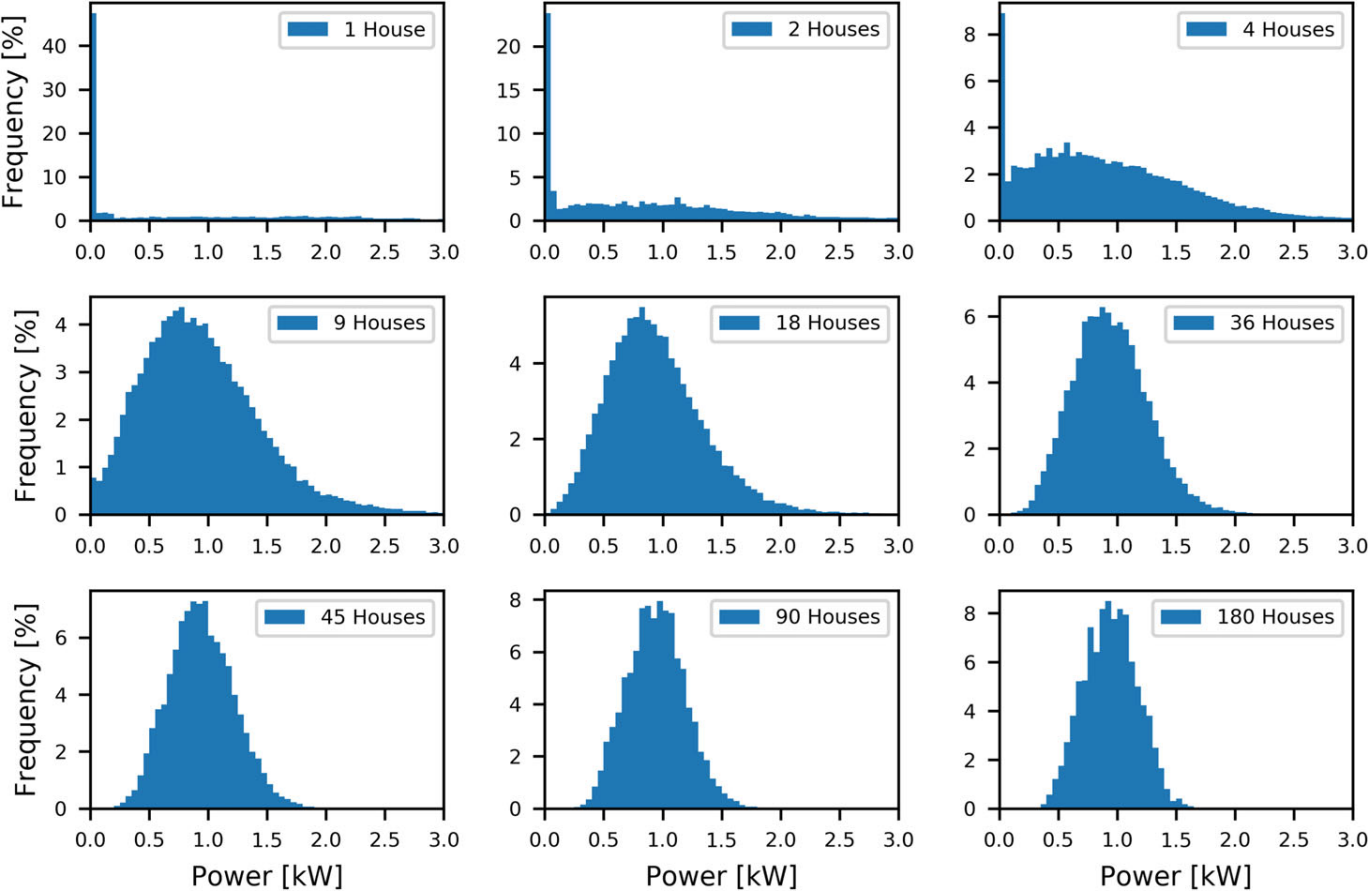
Distributions (bins of 50 W) of the average space heating and hot water power consumption (over 15 minutes) of 180 households grouped in pools of different sizes in January 2020. All histograms were cut at a maximal power of 3 kW to facilitate the comparison. However, for really small pools, it results in part of the distribution not shown

The power consumption of space heating and hot water appliances in a single household is zero most of the time. However, when combining several households, the likelihood of a null consumption for a given time is decreasing. Figure 7 shows the distributions, using bins of 50 W, of the average heating (space and hot water) power consumption (over 15 minutes) of 180 households grouped in pools of different sizes for January 2020, where consumption is dominated by space heating consumption. The households were grouped in pools of 1, 2, 4, 9, 18, 36, 45, 90 and 180, respectively.

Since all 180 households are considered for each histogram, all histograms on Fig. 7 share the same mean value, respectively. Indeed, let us consider *m* pools of *n* households each. The product *m*×*n* equals 180 and the mean power of pool *k* is given by:

$$ \bar{P_{k}} = \frac{1}{n}\frac{1}{q}\sum_{i=1}^{n}\sum_{j=1}^{q} P_{{ijk}} $$

where *q* is the number of quarters of hour in January 2020 (2976). Similarly, the average power of all pools is given by:

$$\begin{array}{*{20}l} \bar{P} &= \frac{1}{m}\sum_{k=1}^{m} \bar{P_{k}} \\ &= \frac{1}{m}\sum_{k=1}^{m}\left(\frac{1}{n}\frac{1}{q}\sum_{i=1}^{n}\sum_{j=1}^{q} P_{{ijk}}\right)\\ &= \frac{1}{m}\frac{1}{n}\frac{1}{q}\sum_{k=1}^{m}\sum_{i=1}^{n}\sum_{j=1}^{q} P_{{ijk}}\\ &= \frac{1}{180}\frac{1}{2976}\sum_{k=1}^{m}\sum_{i=1}^{n}\sum_{j=1}^{q} P_{{ijk}} \end{array} $$

The mean value of all pools is therefore independent of both the number of pools and the number of houses per pool.

**Fig. 8:**
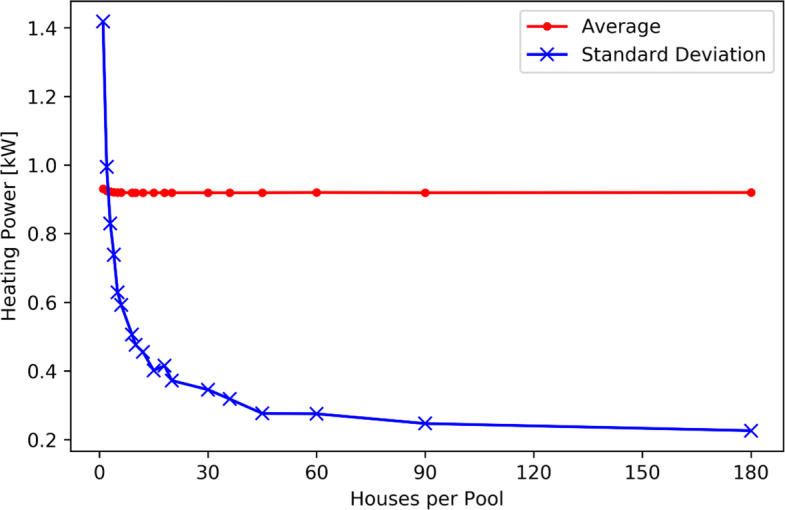
Average and Standard Deviation of the distributions of the average space heating and hot water power consumption (over 15 minutes) of 180 households in January 2020

Figure 7 shows that when the 180 households are considered individually (180 pools of 1 household), the average power during a quarter of an hour is zero most of the time. On the contrary, when increasing the size of the pools, the distribution progressively tends towards a normal distribution with a decreasing variance. This is detailed in Fig. 8 showing the average and standard deviation of the distributions of the average space heating and hot water power consumption (over 15 minutes) of 180 households for January 2020. The households were grouped in pools of 1, 2, 3, 4, 5, 6, 9, 10, 12, 15, 18, 20, 30, 36, 45, 60, 90 and 180, respectively. Figure 8 shows the constancy of the mean values with an increasing number of households per pool, about 0.95 kW for January 2020 (domination of space heating). Figure 8 shows a rapid decrease of the standard deviation with an increasing number of households per pool.

Based on the distributions of Fig. 7, the prediction of the consumption of a large pool of households will be more accurate than the prediction of the consumption of a single household. Considering pools of a sufficient number of households rather than individual households also guarantees that a minimum level of power is consumed at any given time. A minimum of 45 houses is needed in this case to have good quality estimation.

### Shifting potential and effects

**Fig. 9:**
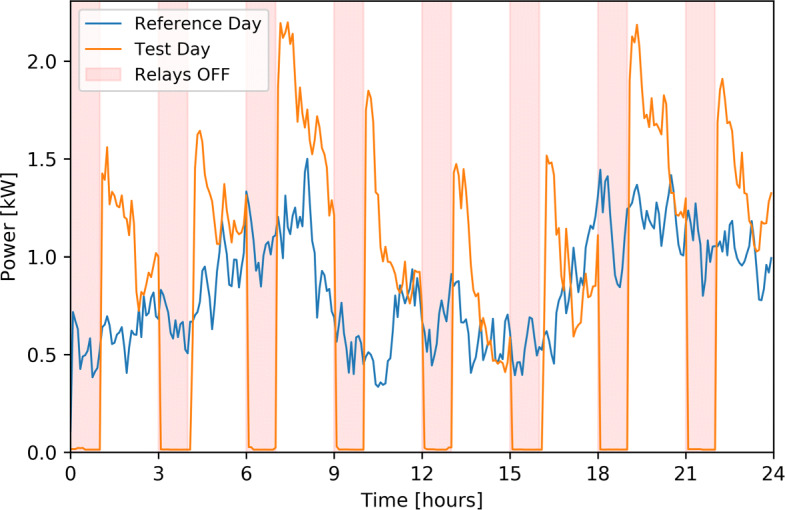
Average heating appliances power of 73 households during a reference day and during a day when regular 1 hour cuts of the appliances were implemented. The rebounding effect is clearly visible as well as a slight oscillation induced by a synchronization of some of the appliances after the cut. The respite period of 2 hours between the cuts seem also to be fitting to restabilize the system after the cuts

**Fig. 10:**
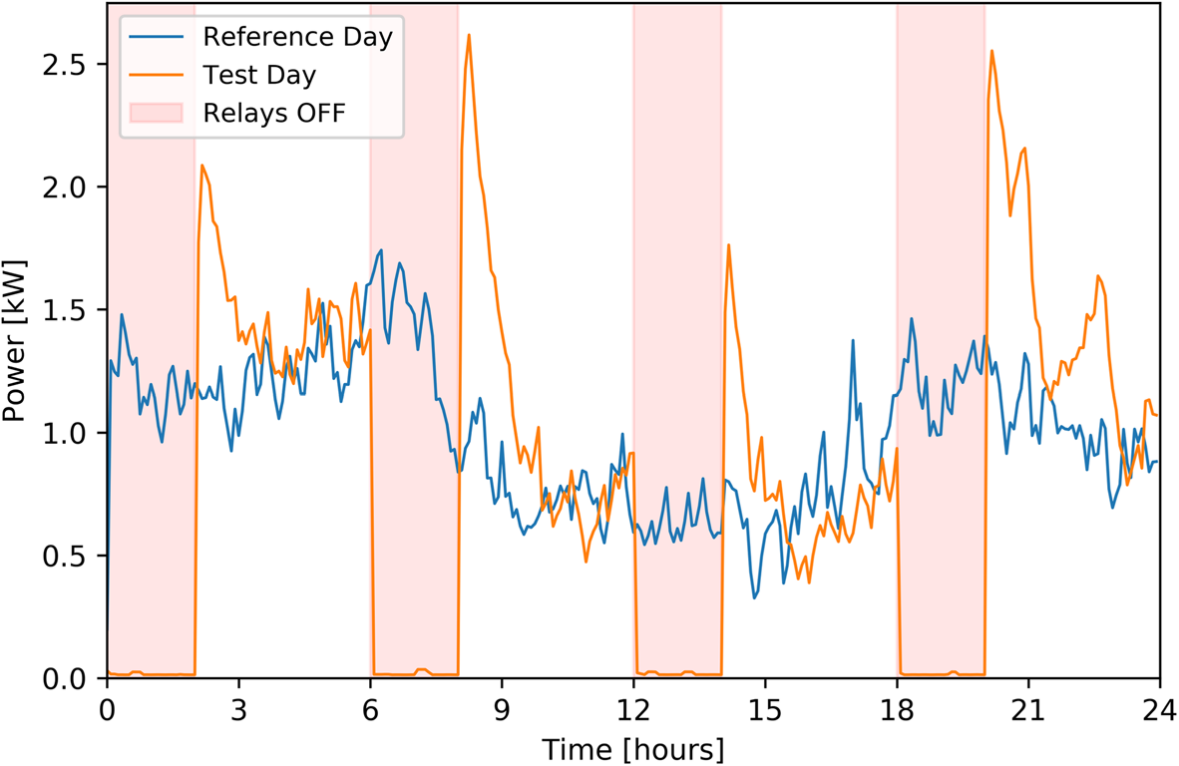
Average heating appliances power of 73 households during a reference day and during a day when regular 2 hours cuts of the appliances were implemented. It can be noted that the rebounds are bigger than with one hour cuts and that the induced oscillations are also more visible

Cut tests of heating appliances were conducted in early spring 2020 for 73 households. The purpose of these tests is to better describe the potential of residential buildings flexibility via studying the behaviour of the loads to be controlled if reacting to flexibility orders. Figure 9 shows the average consumption of heating appliances for all households over a day where cut phases of 1 hour each were executed repeatedly with a two hour resting period versus an average consumption profile of a very similar day (same week) without any cuts. Figure 10 shows the same results while the cut phases are two hour long with a four hour resting period. It should be noted that these cuts of 1 or 2 hours had no impact on the comfort of the inhabitants. Longer periods of cuts (> 2h) could be implemented, but do not seems to be required by the current grid flexibility needs.

When switched off for one hour, heating appliances tend to consume a lot more than usual in the first following hour, to compensate for the removed energy consumption. The consumption gradually decreases for the following hour until reaching the usual consumption. These expected rebound phases tend to be higher in early morning and in the evening, when the average consumption is higher. Their height depends on the erased energy in the previous hour. During the afternoon, when consumption is usually moderate, the rebound effect is shorter in time. It can also be noted that an oscillation is also visible after the first peak. As a good number of appliances will restart exactly at the same time and their on-off period is similar, this synchronization has an impact after the first peak. It is clearly visible in Fig. 10 with the last cut inducing a second peak at 22-23 PM. This oscillation can also be observed after the other cuts in a reduced way. The two hour cuts show that heating appliances consumption tends to stabilize at the reference value after a longer resting period. It should be noted that the rebounding effect could be attenuated by gradually turning appliances back on.

## Conclusion

In this paper, we presented results of tests to quantify households flexibility. They showed that there is a high potential for heating appliances especially in winter for space heating. Hot water heating appliances have a small potential, but more regularly spread over the year. Cut tests showed the possibility to shift heating consumption without altering the comfort of consumers. Flexibility potential was predicted using a model based on temperature and is expected to include more weather parameters in order to have more accurate results. The effect of pooling households on the flexibility prediction was also demonstrated. Further research is conducted to validate this new solution and enhance the flexibility prediction models. The results are expected to be deployed among more households in future years.

## Data Availability

The data used to produce this paper was collected during the GOFLEX trial period. The data is however not openly available.
